# Coastal Habitats as Surrogates for Taxonomic, Functional and Trophic Structures of Benthic Faunal Communities

**DOI:** 10.1371/journal.pone.0078910

**Published:** 2013-10-22

**Authors:** Anna Törnroos, Marie C. Nordström, Erik Bonsdorff

**Affiliations:** Åbo Akademi University, Department of Biosciences, Environmental and Marine Biology, Turku, Finland; James Cook University, Australia

## Abstract

Due to human impact, there is extensive degradation and loss of marine habitats, which calls for measures that incorporate taxonomic as well as functional and trophic aspects of biodiversity. Since such data is less easily quantifiable in nature, the use of habitats as surrogates or proxies for biodiversity is on the rise in marine conservation and management. However, there is a critical gap in knowledge of whether pre-defined habitat units adequately represent the functional and trophic structure of communities. We also lack comparisons of different measures of community structure in terms of both between- (β) and within-habitat (α) variability when accounting for species densities. Thus, we evaluated *a priori* defined coastal habitats as surrogates for traditional taxonomic, functional and trophic zoobenthic community structure. We focused on four habitats (bare sand, canopy-forming algae, seagrass above- and belowground), all easily delineated in nature and defined through classification systems. We analyzed uni- and multivariate data on species and trait diversity as well as stable isotope ratios of benthic macrofauna. A good fit between habitat types and taxonomic and functional structure was found, although habitats were more similar functionally. This was attributed to within-habitat heterogeneity so when habitat divisions matched the taxonomic structure, only bare sand was functionally distinct. The pre-defined habitats did not meet the variability of trophic structure, which also proved to differentiate on a smaller spatial scale. The quantification of trophic structure using species density only identified an epi- and an infaunal unit. To summarize the results we present a conceptual model illustrating the match between pre-defined habitat types and the taxonomic, functional and trophic community structure. Our results show the importance of including functional and trophic aspects more comprehensively in marine management and spatial planning.

## Introduction

The great challenge facing conservation today is how to include the many aspects of biodiversity and ecosystem functioning when establishing and managing protected areas and reserves. The technical innovations in sensing and mapping of the environment give researchers and managers the means to overcome the practicalities of this issue. However, as there always will be costs and limitations associated with gathering data, the use of different surrogate estimates and proxies is growing. These are aimed to encompass the variety of biological diversity, from genes to ecosystems [1]. A surrogate variable approximates the less easily quantifiable measure of interest that would otherwise remain unknown, e.g. the overall richness of species in an area [2]. This means that the efficiency of the surrogate or the proxy must be measurable in terms of how well it correlates with the target feature in space and time, and how well it performs when used for selecting new protected areas [2]. The surrogate can be biotic, such as other taxa [3–5], or abiotic and thus refer to environmental variables [6,7]. An increasingly utilized proxy for biodiversity is some type of pre-defined or classified landscape patch or habitat [8]. Habitats as units have proven popular since they are recognizable and separable in nature based on physical-biological structures such as vegetation types, or abiotic attributes like topography, sediment characteristics or hydro-chemical variables. Thus, mapping of these on a large scale is fairly easily done [9,10]. The use of habitats as surrogates for species diversity have been a given choice in both terrestrial and marine systems because of the extensive and rapid global habitat change which directly affects these ecological units and is one of the most dramatic and clearest consequence of human activities [11–14]. 

In marine systems, taxa as surrogates for taxonomic diversity have been used in a wide variety of habitats [5]. Habitats as surrogate units have on the other hand mostly been applied in and assessed for tropical coral reefs and adjacent ecosystems [8,15–17]. However, habitats have recently also been used to describe the functional structure of communities and particularly fish assemblages [15,17]. The suitability of habitats as proxies for faunal functional structure is usually assessed through determining number of functional groups composed of generally three organism traits (trophic group, size and mobility) [17]. More advanced measures such as functional indices or multitrait analysis have to our knowledge not been used to evaluate the surrogates, although recent theoretical and empirical progress in this field has been rapid [18–21]. Evaluation of functional characteristics is central, as functional divergence between communities in two habitats is not automatically evident from taxonomic composition, which theoretically can deviate substantially without affecting the functional or trophic diversity [22].

The use of surrogates is also on the rise for European temperate benthic ecosystems, as more comprehensive and univocal marine benthic habitat maps and classification schemes (e.g. EUNIS) have been produced [23–26]. Thus, at this stage of the development of benthic marine conservation, asking what actually is conserved when a certain habitat or biotope is safeguarded and managed is not irrelevant. This type of surrogate unit for prediction and planning is to a large extent the level on which future marine environmental legislation and management are set (e.g. EU Habitats Directive1992/2007, EU Water Framework Directive 2000, EU Marine Strategy Framework Directive 2008). Apart from the fact that the accuracy of habitats as proxies for functional structure of communities has rarely been assessed empirically, there are other general gaps in surrogacy and theoretical literature. It is unclear what constitutes an appropriate unit on a community level for conserving trophic structure in nature. Although functional diversity has been taken into consideration to some degree, the functioning of an ecosystem is also dependent on the linkages between and within its components, which relates to food web structure [27]. This aspect of biodiversity and ecosystem functioning adds an important level of complexity and is acknowledged as a critical criterion for implementing a sound and holistic ecosystem-based management [28]. If habitat maps and habitat definitions are going to be used as tools for managers, scientific knowledge is also required about how the trophic structure corresponds to these. The knowledge about the relationships between trophic structure and functioning in marine systems has evolved but has mainly been focused on effects of species loss on different trophic levels and groups [29,30]. Recently, the habitat concept and trophic measures have also been discussed from a more applied perspective [31–34]. Examining stable isotope signals, especially carbon (δ^13^C), of both producers and consumers makes it possible to establish the origins and flow of organic matter that incorporates into food webs in different environments [27]. Spatial differences in consumer isotopic signatures may be due to site-specificity in diets or spatial variability in the signal of the same food source [27]. Studies have shown that isotopic composition of faunal organisms on larger scales, i.e. over hundreds of meters, often show a homogenous pattern due to water movement of particulate organic matter [32]. In comparison, consumers seem to assimilate carbon on much finer scales, within meters, and thus within habitats rather than on a gradient across habitat boarders [33,35]. The question for managers and spatial planners would then be whether the isotope signatures correspond with the pre-defined and mapped habitat units. If this is not the case then either a re-assessment of the precision of habitat maps or a rethinking of suitable units to safeguard trophic structure is needed.

Another related issue is the lack of comparisons of how well pre-defined habitat units simultaneously cover the three focal aspects of community structure: taxonomic, functional and trophic structure. In fact, the relationship between habitat attributes and functional or trophic variables can differ from relationships found for species diversity or compositional patterns [17,36]. Contrasts between specifically taxonomic and functional structures in relation to different environmental variables have been shown in previous work [37–40]. Similarly, taxonomic and trophic structures have shown to vary differently on large and small spatial scales [41]. Therefore, as pointed out above, simultaneous quantifications of all three measures are preferred [36]. This applies especially to marine systems for which more sophisticated marine spatial planning efforts (e.g. Integrated coastal zone - and Ecosystem-based management) and applications of habitat-classification frameworks are on the way [26].

In this study, we are not trying to re-define the habitat concept or definitions, rather test generally accepted types of “habitats” which are easily delineated in nature and defined through classification systems. Instead, our focus is to re-assess the surrogate units when accounting for species density. Variation in organism abundances between habitats is implicit, due to different resource requirements of species. The importance of abundance for understanding habitats in a functional and trophic perspective is not well studied, nor properly considered in marine management [37]. Since the effectiveness of identifying community structure through habitat units is also dependent on the variability among patches of the same habitat, we do not only focus on between-habitat (β) differences but also on within-habitat (α) heterogeneity [16,42].

The general objective of this study was to evaluate *a priori* defined coastal habitats as surrogates for (i) traditional taxonomic, (ii) functional and (iii) trophic zoobenthic community structure. More specifically, we sought to do this by 1) determining between and within-habitat differences for four habitats (sand, canopy-forming algae, and above- and belowground of a mixed seagrass meadow) using univariate community data. We used data on species richness and density as well as functional trait data covering number of traits expressed and species richness within traits. An initial assessment of trophic habitat differences was accomplished by comparing variability in isotope signals between habitats. Furthermore, to comparatively test the efficiency of the surrogates when accounting for species density, 2) we used the subsequent multivariate data on traditional taxonomic composition, functional trait expressions and stable isotope ratios (^13^C:^12^C and ^15^N:^14^N) of benthic primary consumers. We hypothesized that the match between habitats and community structure would vary between the different measures leading to the importance of within-habitat variability. The best match, separate units in all four habitats, was expected for the taxonomic structure since this is to a large extent the information on which we base habitat categorizations. A less accurate match was predicted for the functional and trophic structure in general because of a lower between-habitat heterogeneity, or in other words higher similarity of habitat units.

## Material & Methods

### Coastal habitats and numerical data collection

Macrozoobenthos was sampled in four coastal habitats; bare sand, canopy-forming algae and above and belowground parts of a mixed seagrass bed (hereafter *seagrass aboveground* and *seagrass belowground*) in a moderately exposed shallow bight in the Åland Islands, Northern Baltic Sea in July 2010. These types of habitats are common in shallow coastal bays worldwide and are especially important for primary and secondary production and as nursery habitats for fish [43]. In the Baltic Sea, they are threatened by general habitat degradation e.g. by habitat fragmentation and loss (seagrass meadow), eutrophication and reduced light levels (canopy-forming algae) or drifting algal mats (both bare sand and vegetation) [44–46]. The habitats and the type of higher hierarchy or setting in which they can be found, a “large shallow bay”, are both valued as important. They provide provisional (e.g. food and other resources), regulatory (e.g. sediment retention, eutrophication mitigation) and cultural (e.g. recreational and aesthetic values) goods and services [43,47]. In addition, the embayment and the habitats have high priority in national and international marine conservation and protection strategies (the EU Habitats Directive, the EU Marine Strategy Framework Directive and the HELCOM BSAP 2007). The study was carried out on private land and the owner gave permission to conduct the sampling on this site.

The rocky shore canopy-forming algal habitat sampled in this study consisted predominantly of bladderwrack (*Fucus vesiculosus* L.) and associated epiphytic algae, as well as annual red and green algae to a small extent. The mixed seagrass meadow was dominated by eelgrass (*Zostera marina* L.), but other angiosperm plant species, such as *Potamogeton* spp.*, Nitella* sp.*, Ruppia maritima* L. and *Ruppia chirrosa* L. were also noted (see 48 for more specific site characteristics). Sampling in the canopy-forming algae and the mixed seagrass meadow was conducted in central parts of the vegetation patches to avoid possible edge effects. The organic content (loss on ignition, %) did not differ between the non-vegetated bare sand habitat and the mixed seagrass meadow (Mann-Whitney *p* = 0.222), although it was lower in the sand (mean 0.20 ± 0.01 SE) compared to the meadow (mean 0.32 ± 0.06 SE). Concerning trophic structure, previous studies have shown a distinct isotopic pattern in the benthic food web in the area, both temporally stable - between and within years - and spatially consistent over kilometers [49,50]. Therefore, we chose to replicate within different habitats, rather than regionally. The canopy-forming algal habitat and the seagrass habitats sampled in this study were separated from the sand habitat with a distance of about 100 meters, and were approximately some 300 meters apart from each other.

In each of the four habitats, macrofauna was quantitatively sampled at five locations with three replicates each. All sampling was conducted through SCUBA diving at a depth of 0.5-2.5 m. The epifauna was sampled with a net-bag in a 25 cm × 25 cm area in the canopy-forming algal belt and in the seagrass aboveground habitat. Infauna in bare sand and seagrass belowground was sampled using a sediment core (10 cm diameter and 15 cm height, total volume 4.71 cm^3^, n = 4) in a 25 cm × 25 cm area. The choice of equipment was to attain a reliable estimate of quantified community structure in each habitat. The sampling techniques enabled us to standardize the faunal densities to volume for each replicate (mean volume ± SE for seagrass aboveground and canopy-forming algae respectively: 0.06 ± 0.01 and 0.68 ± 0.08 dm^-3^) rather than 1m^2^. The latter is not a comparable measure to use between these types of habitats due to different sampling methods and physical structure of the habitats. Faunal samples were sieved (0.5 mm) in the field and later stored in 70 % ethanol. Taxonomic resolution was set at species level when possible, or in accordance with the available resolution of trait information. Organisms were collected for stable isotope analysis within three days of the quantitative sampling, to minimize the possible mismatch between habitat-specific community patterns and effects of temporal change in isotopic values [49].

### Biological traits

Biological traits of benthic species were applied based on previously gathered information in a trait dataset of northern Baltic Sea macrozoobenthos [51]. This trait dataset is publicly available as a supplement to [51].The information in the trait dataset was collected on lowest possible taxonomic level and only later adjusted to comparable higher taxonomic levels. Thus, if species/genera displayed differing expressions, the taxon was given equal probabilities of expressing the trait modality. The information was gathered and used based on i) peer-reviewed published sources, ii) phenotype or other species-specific characteristics, or iii) expert knowledge [51]. The same standards regarding trait information were applied in this study. An exception regarding trait information in this study was the trait Mean Size, which here refers to species-specific individual lenght measurements for each habitat (mean size per habitat, measured under a light microscope to nearest mm). The methodology of assigning traits follows the categorical approach where a trait (e.g. Mobility) is divided into sub-categories or “modalities” (e.g. sessile, semi-mobile or mobile). Species are then scored using a fuzzy coding procedure [52], so that they express each modality on a scale from 0 to 3. These scores are then standardized to 1 within traits. This coding procedure accounts and allows for species plasticity in expressing traits. For assessment of functional differences within and between the habitats, we used 13 traits, encompassing a total of 55 modalities (Table 1). Our rationale for choosing traits was to adequately capture a broad set of different taxon characteristics across the macrofaunal community, which can depict differences between habitats. 

**Table 1:** Traits and modalities included in the study.

**Trait**	**Modality**
Mean Size	1-5mm
	5mm-1cm
	1-3cm
	3-5mm *
	>5cm
Longevity	Very short
	Short
	Long
	Very long
Reproductive technique	Asexual
	Sexual
Sexual differentiation	Gonochoristic
	Hermaphrodite
	Parthenogenetic *
Developmental technique	Fragmentation
	Oviparous
	Ovoviviparous
	Viviparous
Reproductive frequency	Semelparous
	Annual episodic
	Annual protracted
Living Habit	Attached
	Tube dweller
	Burrow dweller
	Case builder *
	Free
Environmental position	Infauna deep (>5cm)
	Infauna middle (2-5cm)
	Infauna top (2cm)
	Epibenthic
	Bentho-pelagic
Feeding habit	Suspension feeder
	Surface feeder
	Sub-surface feeder
	Selection feeder
	Miner
	Parasite *
Resource capture type	Jawed
	Siphon
	Tentaculate
	Pharynx
	Radula
	Net
Mobility	Sessile
	Semi-mobile
	Mobile
Movement type	Swimmer
	Rafter-drifter
	Crawler
	Byssus threads
	Tube
	Burrower
Dispersal habit	Non dispersal
	Local
	Long-distance
**TOTAL: 13**	**55**

A total of 13 traits and 55 modalities were used in the multivariate (abundance-weighted) analysis. All modalities were expressed in the canopy-forming habitat. The following four modalities indicated with an asterisk (*) were not expressed in all habitats: 3-5mm (sand, seagrass belowground), parthenogenetic (sand), case builder (sand, seagrass above- and belowground), parasite (sand).

To compare the functional approach to the two others, the information on faunal densities in each habitat were combined with the respective trait scores. In this procedure, trait scores were multiplied by species abundance and then summed over all species in each replicate in a habitat. This habitat-by-trait matrix was used for both univariate and multivariate analyses on differences in trait expressions within and between habitats.

### Stable isotope analysis

To assess differences in isotope signatures between the habitats and, as a first step, portray the principal food web structure, we sampled sediment organic matter, suspended organic matter, primary producers (macrophytes and associated flora) as well as primary consumers (macrofauna). Organic matter from the sediment and the seawater was sampled in the bare sand habitat, from the top 2 cm of the seafloor and with a 10 μm mesh net, respectively. Both types of organic matter were collected on pre-combusted (500 C, 4h) Whatman GF/C glass fiber filters, which were dried, acidified with 1 N HCl and dried again before cut into pieces and packed into tin capsules. Macrophytes were rinsed in distilled water and cleared of all associated biota by hand. Epiphytes were collected from *F. vesiculosus* and *Potamogeton pectinatus*. Infaunal and epifaunal organisms were determined to the lowest taxonomic level possible (Table 2). The material was stored in -20°C before being treated with HCl, oven-dried in 60°C (48h), and ground to a fine homogenous powder, after which an aliquot of the sample was packed in a tin capsule. All samples consisted of several whole individuals. Mollusc samples consisted of soft tissue, removed from the shells. The analysis of stable isotope ratios was conducted at the Stable Isotope Facility, UC Davis. Carbon and nitrogen isotope ratios are reported in units of per mille (‰) delta (δ) values according to:

δ^13^C or δ^15^N = [(R_sample_/R_standard_ - 1)] x 10^3^ (1)

where R is ^13^C:^12^C or ^15^N:^14^N. The standard for carbon is Vienna-PDB and for nitrogen atmospheric N_2_ [53]. We analyzed three and five replicate samples per species per habitat for macrofauna and macrophytes, respectively.

**Table 2:** Benthic macrofaunal species found in the sampled habitats.

**Phylum**	**Group (Order/Class)**	**Species**	**Habitats**			
			**Sand**	**Canopy-forming algae**	**Seagrass above-ground**	**Seagrass below-ground**
Nemertea	Enopla	*Cynopthalma obscura* (Schultze)	X	X	X	X
Priapulida	Halicryptomorpha	*Halicryptus spinulosus* (von Siebold)	X			
Annelida	*Oligochaeta*	*Oligochaeta*	X	X	X	X
	Hirudinea	*Piscicola geometra* (Linnaeus)		X	X	X
	Polychaeta	*Marenzelleria* spp. (Mesnil)	**X**	X	X	**X**
		*Boccardia* (*Polydora*) spp. (Horst)	X			
		*Pygospio elegans* (Claparide)	**X**	X	X	X
		*Hediste* (*Nereis*) *diversicolor* (O.F.Möller)	**X**		X	**X**
		*Blygides* (*Harmothoe*) *sarsi* (Malmgren)		X		
		*Manayunkia aestuarina* (Bourne)			X	X
Mollusca	Gastropoda	*Hydrobia* spp. (Hartmann)	X	X	**X**	X
		*Potamopyrgus antipodarum* (Gray)		X	X	X
		*Radix* spp. (Montfort)		X	**X**	**X**
		*Bithynia* sp. (Leach)			X	
		*Theodoxus fluviatilis* (Linnaeus)	X	**X**	**X**	**X**
	Bivalvia	*Cerastoderma glaucum* (Poiret)	X	X	**X**	**X**
		*Macoma balthica* (Linnaeus)	**X**	X	X	**X**
		*Mya Arenaria* (Linnaeus)			X	**X**
		*Mytilus edulis* vel. *trossulus* (Linnaeus)	X	**X**	**X**	**X**
Crustacea	Isopoda	*Idotea balthica* (Pallas)		**X**	**X**	**X**
		*Idotea chelipes* (Pallas)	X	**X**	**X**	**X**
		*Idotea granulosa* (Pallas)		X	X	
		*Jaera* spp. (Leach)	X	X	X	X
		*Saduria entomon* (Linnaeus)				X
	Amphipoda	*Gammarus* spp. (J.C.Fabricius)	X	**X**	**X**	X
		*Calliopius laeviusculus* (Kröyer)			X	
		*Leptocheirus pilosus* (Zaddach)		X		
		*Monoporeia affinis* (Lindström)		X		
		*Bathyporeia pilosa* (Lindström)	**X**	X		
		*Corophium volutator* (Pallas)			**X**	X
	Mysidae	*Mysis mixta* (Latreille)		X	X	
		*Praunus flexuosus* (O.F.Möller)		X	X	
Insecta	Coleoptera	*Macroplea mutica* (larvae) (Fabricius)			**X**	X
	Diptera	*Chironominae* spp.	X	**X**	X	**X**
		*Tanypodinae* spp.		X		
		*Orthocladiinae* spp.		X	X	
		**TOTAL: 36**	17	26	29	23

Species indicated with an X in bold were included in the stable isotope-analyses.

In order to quantitatively compare the ability of the surrogates to target all three measures of community structure, we also weighted the isotopic values of the primary consumers with the habitat-specific abundance data, producing a habitat-by-stable isotope matrix.

### Statistical analysis

We used generalized linear models for all univariate analyses of between and within-habitat differences for the taxonomic and functional approach. This type of analysis allows for various sample distributions [54]. Our data, being count data and standardized between habitats, was after transformations still non-normally distributed or showed heterogeneous variances, whereby generalized linear models were appropriate. A nested analysis with ‘Location’ (5 levels) nested within ‘Habitat’ (4 levels) was run in SPSS v. 18.0 on both traditional community variables (species richness and abundance) and functional variables (No. of modalities and mean No. of species per modality). For species richness and density we used a Poisson and a Gamma distribution as the sample distribution, respectively. For the functional variables a normal distribution fitted the data best. The model fit was evaluated for each data set by examining the ratio of the deviance to its degrees of freedom, for which a value close to one indicates good fit [54]. Outliers were also considered and one replicate in Seagrass aboveground was removed, considered to be a true outlier due to a laboratory handling error. Further, we checked the normality of the residual variation. We chose a logarithmic (species richness), power of two (mean abundance) or an identity link function (No. of modalities, mean No. of species per modality) to transform and connect the mean of the dependent variable and the linear combination of the predictor variables [54,55]. This type of statistics is based on the maximum likelihood estimation and tests of the chi-square distribution.

To assess principal habitat differences in consumer community isotopic niches, we used a one-way PERMANOVA on δ^13^C and δ^15^N values of benthic macrofauna. A similar but separate analysis was applied on the two sources of organic matter and the primary producers.

Multivariate analyses were also used to depict and assess habitat differences in taxonomic composition (traditional abundance weighted habitat-by-species data), functional trait expression (abundance weighted habitat-by-traits data) and trophic composition based on isotope values (abundance weighted habitat-by-stable isotope data). For the first two data sets, a nested PERMANOVA with ‘Location’ (5 levels, random) nested within ‘Habitat’ (4 levels, fixed)) was run to determine between and within habitat differences, which were visualized using MDS (based on Bray-Curtis similarity) with a cluster overlay. Since one outlier was removed, the design was unbalanced. This issue was checked, according to procedures for unbalanced designs with PERMANOVA [56], and corrected using the conservative Type III sum of squares. A one-way PERMANOVA analysis (factor: ‘Habitat’ (4 levels, fixed)) was run on the abundance weighted habitat-by-stable isotope matrix. The result was visualized according to the other multivariate analysis on abundance-weighted data, using a MDS (Bray-Curtis similarity) with a cluster overlay. Permutational tests of multivariate dispersion (PERMDISP, [57]) were used alongside all PERMANOVA analyses to check for homogeneity in average dispersion of samples from their group centroids. Prior to the analyses using density data, abundance values were square root transformed. To identify contributions of species and traits to possible within and between habitat differences, SIMPER analyses were used [58]. All multivariate analyses were run in PRIMER v.6.

## Results

### Habitats as surrogates - basic differences in community structure

As we expected, a difference between all four habitats was found for both the taxonomic and the functional structure when assessed with the univariate statistics. Both species richness (Habitat: df = 3 X^2^ = 70.26 *p* = 0.001) and density (Habitat: df = 3, X^2^ = 28.52 *p* = 0.001) differed between habitats, with highest species richness and total abundance found in the seagrass aboveground habitat (29 spp. in total, and mean abundance 5974.7 ± 4154.3 dm^-3^) and lowest on bare sand (17 spp. in total, and mean abundance 18.4 ± 11.5 dm^-3^). In total, 36 macrofaunal taxa were found in the habitats (Table 2). Thus, no habitat by itself hosted all species (Table 3). Generally, about one fourth (10/36 spp.) of the species were only found in a single habitat and about as many species occurred in all habitats (13/36 spp.), indicating an equal proportion of rare and common species in the habitats (Table 3). In terms of number of modalities, the effect of within-habitat variability could not be disregarded for understanding between-habitat differences (Location(Habitat): df = 16 X^2^ = 42.45 *p* = 0.001). In general, the seagrass above- and belowground showed the highest number of modalities (average 50.6 ± 3.4, average 51.7 ± 1.1, respectively). In comparison to species richness, which varied between habitats, almost all trait modalities were expressed in all habitats (Table 3). The modality, *case builder* within the trait Living habit, was unique and only found in one habitat: the canopy-forming algal habitat. The body size modality *3-5mm* was also rare and only found in the epifaunal habitats. In addition, the modalities *parthenogenetic, sexual differentiation* and *parasitic* were found in all other habitats but the sand habitat (Table 1). The habitats were also distinct when it came to the average number of species per modality (Habitat: df = 3 X^2^ = 378,83 *p* = 0.001, Location(Habitat): non-significant *p* = 0.630). Interestingly, even though the canopy-forming algal habitat showed higher species richness than the seagrass belowground (26 spp. vs. 23 spp.), the average number of species per modality was still higher in the less diverse habitat (3.7 spp./mod. compared to 4.2 spp./mod. respectively) (Table 3).

**Table 3:** General numerical and functional descriptives of the macrofaunal communities in the habitats.

**General descriptives**	**∑ Tot.**	**Sand**	**Canopy-forming algae**	**Seagrass aboveground**	**Seagrass belowground**
***Traditional numerical variables***					
# of species	36	17	26	29	23
# of species only in one habitat	10	2	4	3	1
# of species found in all habitats	13				
Abundance dm^-3^ (Mean ± SD)		18.4±11.5	2733.8±1624.8	5974.7±4154.3	110.0±44.2
***Functional variables***					
# of modalities expressed (Total)	55	51	55	54	53
# of modalities (Mean ±SD)		44.3±3.1	48.5±1.8	50.6±3.4	51.7±1.1
# of species per modality (Mean)		2.4	3.7	4.5	4.2

Results are given as total number if not indicated otherwise.

Contrary to the results above, no habitat differences were identified for the principal trophic pattern of primary consumers based on the isotope ratios (one-way PERMANOVA: *p* = 0.241). The species included in the isotope analysis quantitatively ranked within the top 10 species. However, there was a true potential for identifying the habitat differences of the consumers since the resource base, i.e. the sediment and suspended organic matter as well as primary producers and associated epiphytes, varied significantly (one-way PERMANOVA: *p* < 0.001). The two sources of organic matter in sand and the macrophytes all showed distinct isotopic values (mean values of δ^13^C and δ^15^N for sediment organic matter: -17.7, 3.3, suspended organic matter: -18.9, 2.3, *F. vesiculosus*: -11.8, 3.4, *Z. marina*: -10.5, 3.5 and *P. pectinatus*: -8.3, 2.8, respectively). Epiphytes also differed from the other sources and showed similar carbon values as the organic matter in the sand habitat but was more depleted in ^15^N (mean values of δ^13^C and δ^15^N for epiphytes on *F. vesiculosus*: -18.5, 1.4 and on *P. pectinatus*: -19.9, 1.9, respectively). This result indicated that all four habitats comprised one common food web from a primary consumer perspective. Thus on this spatial scale of hundreds of meters without factoring in faunal density, none of the pre-defined habitat units could serve as a surrogate for trophic structure.

### Comparison of taxonomic, functional and trophic structure in pre-defined habitat types

In agreement with the univariate analyses, the habitat units also reflected distinct taxonomic and functional multivariate community structures (nested PERMANOVA: *p* < 0.001 and *p* = 0.028 respectively) (Table 4). The PERMDISP analyses showed the dispersion of samples to be homogenous between groups for both species and trait compositions (Location: *p* = 0.970 and *p* = 0.969, respectively). The two measures also showed the same pattern of variation within habitats, among sampling locations. Differences within habitats were not general to all habitats and subsequent pairwise comparisons showed this to only be an effect in the canopy-forming algal habitat. Within this habitat, the taxonomic composition varied between two pairs of stations and the functional composition within one pair of stations. In addition, since the greatest variation was found on the level of habitats and not location, in both the taxonomic and the functional PERMANOVA analysis (square root of the estimated component of variation due to the habitat 53.1 and 46.4 respectively), the between-habitat difference was believed to be valid, despite this within-habitat difference. The comparative trophic analysis, which accounted for density in the trophic structure, showed significant habitat differences contrary to the preliminary trophic analysis that used simple stable isotope ratios. However, these differences did not reflect the *a priori* habitat divisions (one-way PERMANOVA: *p* < 0.001, PERMDISP analysis: *p* = 0.492) (Table 4). The Post hoc tests showed that a separation was found between the epifaunal (canopy-forming algae and seagrass aboveground) and the infaunal (bare sand and seagrass belowground) habitat units, not between all of the habitats as was the case using the two other multivariate measures (canopy-forming algae vs. seagrass aboveground: *p* = 0.730 and sand vs. seagrass belowground: *p* = 0.939).

**Table 4:** PERMANOVA results for traditional, functional and trophic measures of habitat differences.

**PERMANOVA (nested or one-way)**	**df**	**MS**	**Pseudo-F**	**P(perm)**
***Traditionalmeasure*: Species composition**				
Habitat	3	42045	48.879	0.0001 ***
Location(Habitat)	16	862.26	1.778	0.0001 ***
Residual	39	484.99		
***Functional measure*: Trait composition**				
Habitat	3	31894	98.024	0.0001 ***
Location(Habitat)	16	325.89	1.419	0.0282 **
Residual	39	229.75		
***Trophicmeasure*: Stable Isotope composition**				
Habitat	3	8153.3	7.823	**0.0002** ***
Residual	23	1.42.3		
Total	26			

All three measures were based on macrofaunal abundance; species abundance, abundance weighted traits or abundance weighted δ^13^C and δ^15^N values, respectively. A nested PERMANOVA design was used for the traditional and the functional measures, while a one-way PERMANOVA design was used for the trophic measure. Significant values are indicated in bold. All analyses were based on 9999 permutations. Sample dispersion was homogenous between groups (PERMDISP all *p* > 0.05).

**Figure 1:**
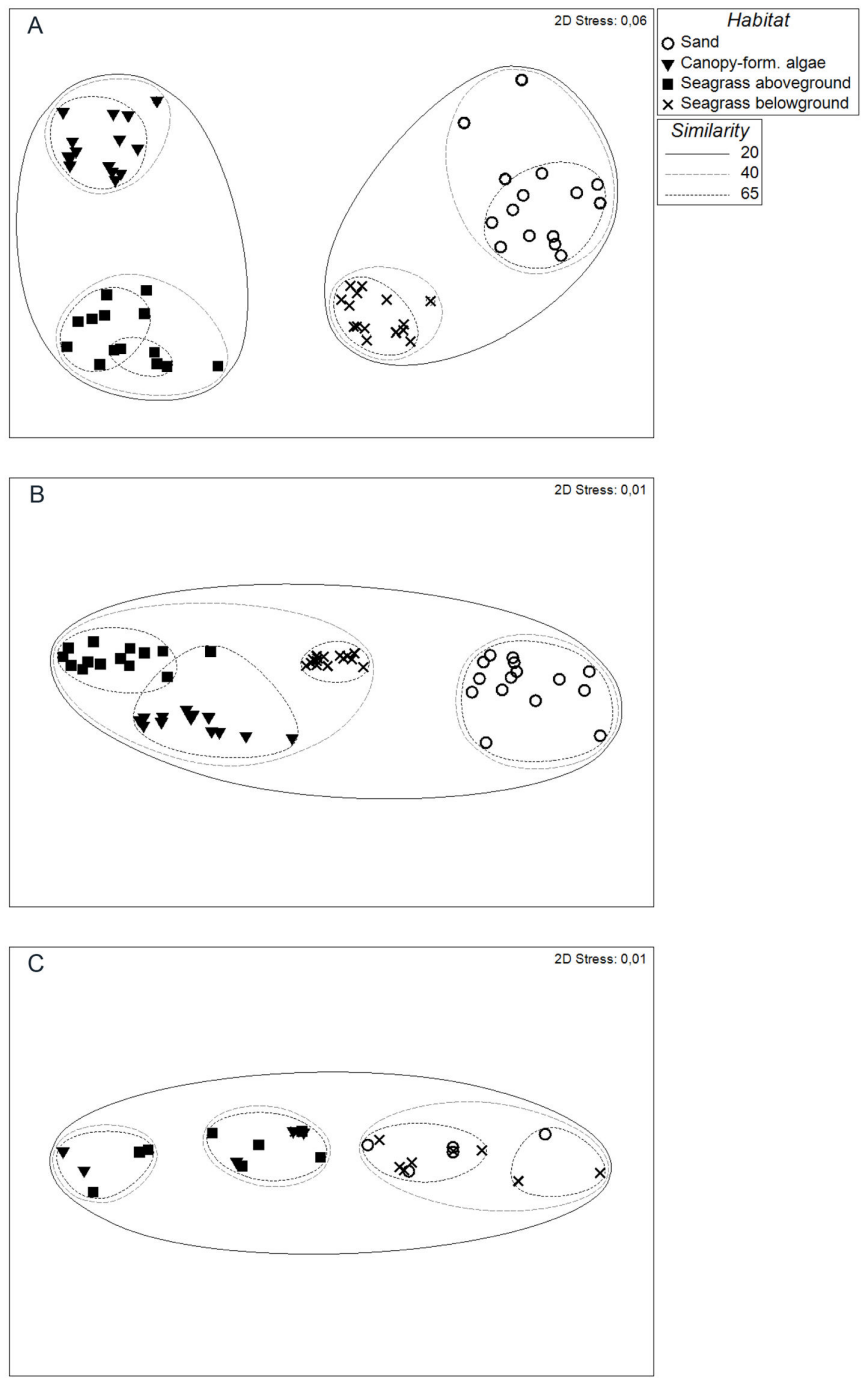
MDS configuration on taxonomic, functional and trophic community structure. MDS configuration with superimposed clustering from Bray-Curtis similarities on a) taxonomic b) functional and c) trophic structure of the macrofaunal community. Groupings of habitats are indicated at three different within-group similarity levels (continuous black line 20 %, dashed line 40 % and dotted line 65 % similarity).

The different outcomes of the PERMANOVA analysis regarding the fit of the habitat types justified a more thorough examination of the role of within-habitat variability for the choosing of the surrogate unit. Though the division of four habitats was significant and followed the same pattern for both the taxonomic and functional measure, the functional structure was on average more similar in terms of between-habitat differences than the taxonomic structure. In fact, the difference between the two data sets in terms of dissimilarity between habitats was on average 29 %. This similarity was specifically reflected in the within-habitat heterogeneity (Figure 1a, b). The lowest within-habitat similarity level on which any grouping in the taxonomic structure could be identified was a Bray-Curtis level of 20 % similarity. On this level, two clusters separated the epifaunal from the infaunal habitat types (Figure 1a). However, functionally and trophically all habitats grouped into one cluster on this level (Figure 1b, c). At a 40 % similarity level, the taxonomic structure was sufficiently homogenous within habitats for all of the four habitats to be identified as separate units (Figure 1a). This was the highest level of similarity within habitats on which the *a priori* habitat division was found taxonomically. Higher similarity levels only illustrated smaller within-habitat clusters. The corresponding functional pattern was a distinction of only two groups: the pre-defined bare sand habitat and a cluster including the three other habitat units (Figure 1b). The trophic structure on this level resembled the taxonomic structure more and separated the epifaunal from the infaunal habitats, in accordance with the PERMANOVA analysis (Figure 1c). Thus, on the same level of within-habitat variability that identified all four habitats taxonomically, functionally and trophically only two groupings could be identified although in slightly different combinations. In order to identify all four units based on trait composition, a 65 % within-habitat similarity was needed (Figure 1b). In other words, when the groups conformed to the *a priori* habitat types, they were within themselves only 35 % dissimilar in terms of trait variability. In comparison, the taxonomic structure showed a dissimilarity of 60 % for the within-habitat variability (40 % similarity level), which was almost twice as high as for the functional structure. The trophic structure was even more heterogeneous on this level and divided the epi- and infaunal groupings further, into four separate clusters (Figure 1c). 

Species that contributed most to the taxonomic differences between and within habitats were all generalists and common in terms of both occurrence and high average density in habitats. These were for example: *Chironominae* spp., *Gammarus* spp, *Hydrobia* spp. and *Macoma balthica*. Modalities that drove the functional differences were predominantly associated with reproduction and living habits such as *sexual reproduction*, *gonochoristic differentiation* (separate sexes) and *annual protracted reproduction* as well as *free* in terms of living habit (SIMPER analysis). However, *mobile* and the size category *1-5mm* were the trait modalities that continuously grouped as top two for contributing to the habitat differences.

## Discussion

**Figure 2:**
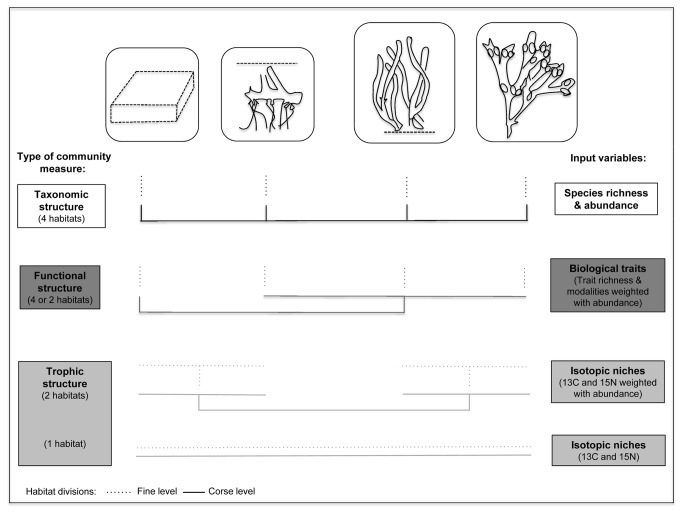
Conceptual model on habitats as surrogates for taxonomic, functional and trophic structure. A conceptual model illustrating the match between pre-defined habitat types and traditional taxonomic, functional and trophic community structure on a coarse and a fine level of habitat division. Habitats as surrogates could be used to safeguard taxonomic and functional structure on a fine level (four separate units). However, high functional similarity is evident on a coarser scale and is important for management to recognize. On this scale, only bare sand as a habitat type is clearly functionally different, and a separation into only two habitat units is possible. Habitat divisions may not meet the variability of the trophic structure at all (based on simple stable isotope values) or only show a distinction between epi- and infaunal habitat types (accounting for density in the trophic structure). Type of community measure (taxonomic, functional or trophic structure) and number of pre-defined habitat types identified using that measure are shown on the left and specific input variables (species density, biological traits or stable isotopes) used in the analyses on the right.

Studies that have assessed habitats as surrogates have focused mainly on effectively safeguarding taxonomic diversity [8,15]. Our aim was to broaden this knowledge and test if traditional habitat divisions also encompass the functional and trophic structures of benthic macrofaunal communities. We found a generally good fit between differences in habitat types and taxonomic and functional structure of the community, even though habitats were considerably more similar functionally than taxonomically. The habitat divisions did not meet the variability of the trophic structure, which proved to differentiate within rather than between the pre-defined habitat units. To summarize these results, we choose to conceptually illustrate the contrasts between the three measures of community structure for a fine and a course division of the habitat units (Figure 2). Using the habitats as surrogates on a fine level, attention should specifically be paid at the measure of trophic structure for which there is potentially only one unit (isotopic niches) or a division between the infaunal and the epifaunal habitats (abundance - weighted stable isotopes). On the other hand, using habitat units on a more coarse level it is noteworthy that only two units might be enough to encompass the functional structure. Thus, the conceptual model illustrate ecologically important issues with using habitats as surrogates that could specifically be of value for marine conservation and spatial planning decisions.

### Coastal habitats as surrogates for multiple community measures?

The results found in this study on the traditionally used measure for surrogates, namely the taxonomic structure, are supported by several empirical studies on diversity of macrofaunal community in the system [59–62]. The taxonomic coherence with habitats is also generally concluded from other marine systems, both temperate and tropical [3]. Thus, the results found here are to some degree applicable on a larger scale. Unsurprisingly, the largest differences for all three measures were found between the structurally complex habitats (canopy-forming algal habitat and seagrass aboveground) and the other two (sand and seagrass belowground). The clear separation among the habitats associated with the sediment, particularly in the functional analysis, was probably also a result of higher structural complexity and sediment stability. This was most likely due to lower hydrodynamic exposure in the seagrass belowground compared to the homogenous bare sand habitat [59]. The importance of using density-based measures for identifying community differences and efficiency of habitat as surrogates was clear from this study, particularly concerning the functional and trophic analyses. Previous studies have also highlighted the role of density rather than specific traits for understanding the functional structure [37]. In this study the abundance-weighted functional analysis demonstrated a similar community *traitscape* for the canopy - forming algal and seagrass habitats. By *traitscape*, we refer to the distribution and composition of biological traits in an area or habitat. Here this corresponds to the clustering together of three habitat types as one functional unit rather than three separate ones on a coarse level of dividing the habitats (Figure 2). Additionally, trophic differences between habitats were only seen when accounting for abundance, not when analyzing stable isotope ratios as such (Figure 2). There could be several mechanisms behind the fact that habitats seemed to be homogenous in terms of trophic and functional structure. The results could be attributed to strong connectivity and metacommunity dynamics between habitats, abiotic movements of nutrients or simply similar food sources (e.g. epiphytes) of species [27]. The similarity of food source was confirmed in our study. However, the importance of consumer plasticity in coastal macrofaunal communities makes it difficult to generalize based on a specific site or habitat [63]. The scale of ecological sampling is always decisive and microhabitat scales have proven to stand for a considerable amount of variation in food web patterns [27,64]. The aim in this study was to compare three measurements of community structure on ecological scales relevant for conservation and spatial planning. The within-habitat scale was, however, the most significant in this study and the stable isotope variability was observed across the whole bay. We argue that the pattern seen in this study is valid and possibly more common than previously thought since previous studies have shown a similar trophic pattern for coastal bays in the region [49,50]. Moreover, the habitat types assessed in this study usually occur in relatively close proximity to each other. Though there are difficulties balancing between the proper scale of measurement and management, we cannot simply disregard the need for marine management efforts that focus on trophic structure and food web dynamics. Instead it could be of advantage to adopt an approach in which especially trophic diversity is safeguarded *across habitats* on a larger scale, rather than *between habitats* on a local scale, e.g. within a bay. On the other hand, when organism densities were accounted for trophically, the distinction found between the epifaunal and infaunal habitats gives some positive indication for relying on the traditional habitat divisions (Figure 2). However, we strongly encourage further assessment of the spatial correlation of trophic structure in nature.

The contrast between the measures of taxonomic, functional and trophic structure and the mismatch between especially the trophic variability and the habitats showed that habitats as surrogates may not be empirically adequate for all types of diversity measures (Figure 2). The terrestrial surrogacy literature states that the chosen set of surrogates must ultimately ensure the full representation of the target parameter more so than spatial correlation and congruence [2]. Accordingly, the habitats in this study could be said to effectively represent, besides the taxonomic, also the functional structure even though the habitat division does not completely match the functional variability on both spatial scales. Particularly noteworthy is the lack of accuracy with the pre-defined habitat units on a within- (α) habitat scale due to the generally high functional similarity (Figure 2). This mismatch does not imply that the functional measure is inaccurate, rather that that it has the potential to illustrate variability on several scales. The importance of this type of low functional beta-diversity despite a simultaneous high taxonomic diversity has recently been highlighted for understanding community assembly and processes structuring communities over different scales [36]. However, this spatial disagreement, probably an effect of the openness of marine systems, is a reason why surrogacy work in marine benthic systems should specifically attend to and tackle this issue [5]. Consequently, planners relying on traditional habitat maps and habitat categorizations may cautiously proceed to do so under the assumption that it captures taxonomic and to some degree also the functional trait diversity. These types of inherent species-related community differences in the habitats are today to some extent incorporated as secondary parameters on lower levels in e.g. the EUNIS habitat classification system [23]. However, including functional aspects more comprehensively in such classifications and in the way habitat maps are constructed is essential for more diverse and targeted future spatial planning. Especially since incorporating a broader knowledge of species traits such as feeding, reproductive, mobility and behavioral ones, a part form the most often used body size, is relevant for understanding and measuring human impacts on benthic communities (e.g. trawling impacts or non-ingenious species invasions) and spatial patterns of benthic functional diversity [65,66]. 

The contrasting outcomes that we present here should not be seen as a strict either-or scenario or conflicting management choices, rather the opposite. A species-centered way of management, when applied strictly on a habitat level, is appropriate in regard to rare, threatened or endangered species [1]. As we show here, it is essential that we also incorporate an understanding of the optimal unit for safeguarding the functionality of the system, both in terms of functional and trophic diversity. We should not continue to rely on deducing such information from the taxonomic structure only because including such units in conservation actions is challenging. Instead, we should aim to assess the management strategies empirically. This could also mean cost-effectiveness in terms of safeguarding the diversity of key functional or trophic hot spots, rather than a multitude of functionally similar habitats. As shown in a previous study, the gain of utilizing the biological trait approach over the traditional taxonomic one resulted in a considerable lower sampling effort, with smaller areal coverage required for understanding functional compared to taxonomic patterns [51].

### Conclusions and future directions

Our study highlights significant discrepancies when using habitats as surrogates for different measures of biodiversity, reflecting the ecological relevance of habitats in terms of taxonomic, functional and trophic structure. For management, the relevance of our findings arises from current mapping activities, habitat classification and spatial planning efforts. These management interests might not target the proper level for safeguarding functional or trophic properties of diversity. We believe future mapping and modeling of marine habitats should not only emphasize traditional taxonomic diversity, but also aim towards assessing distributions of functionally different units in coastal areas. Knowledge of specific processes such as energy- and elemental transfer between different coastal habitats would benefit from comparisons among biomass-based measures of community structure. In relation to this, the important question for managers and policy makers is what we stand to lose functionally if a certain habitat is lost? As shown in this paper it could be valid to disregard traditional habitat divisions in favor of safeguarding the functioning in a landscape or region. In this specific study, this could have meant conserving any type of aboveground habitat, (either algal or macrophyte) and the bare sediment. Such choices are based on the habitats forming distinct functional and trophic units. It is evident that there is a role for biological trait analysis in assessing trophic and functional relationships, as well as in marine management schemes, however the latter still requires proper protocols and assessments [67].
